# Transcriptomics and resequencing reveal dual evolutionary strategies for salt tolerance in foxtail millet (*Setaria italica* (L.))

**DOI:** 10.3389/fpls.2026.1938267

**Published:** 2026-09-16

**Authors:** Min Liu, Ao Shen, Fu-Gang Xin, Ru-Mei Tian, Guan Li, Shuang Dai, Song Hou, Xiao-Ming Wang, Guang-Yu Fan, Shi-Lu Cao, Wei Liu

**Affiliations:** 1 National Medium-Term Genebank for Drought-Resistant and Saline-Alkali Tolerant Crop Germplasm Resources (Jinan), National Saline-Alkali Tolerant Crop Germplasm Resources Nursery (Dongying), Institute of Crop Germplasm Resources, Shandong Academy of Agricultural Sciences, Jinan, China; 2 Zhangjiakou Academy of Agricultural Science, Zhangjiakou, China

**Keywords:** dual evolutionary strategy, foxtail millet (*Setaria italica*), salt tolerance, selective sweep, transcriptional plasticity, WGCNA

## Abstract

**Introduction:**

Soil salinity poses a major constraint to cereal production, yet the genetic basis underlying salt tolerance in foxtail millet (*Setaria italica* (L.)) remains largely elusive.

**Methods:**

To dissect these tolerance mechanisms, we integrated phenotyping, physiological assays, transcriptomics, and population genomics across 18 extreme salt-tolerant panel (STP) and 18 salt-sensitive panel (SSP) accessions selected from over 500 germplasm resources, with evaluations conducted under NaCl treatment.

**Results:**

Under salt stress, STP accessions exhibited better growth and higher chlorophyll content (RCC: 5.9 vs. 3.0 on day 2) alongside lower membrane damage (REC: 14% vs. 24%) and ROS accumulation compared to SSP accessions. Transcriptome profiling identified a tolerance-associated module, STP007, which was enriched for AP2/ERF and IDD motifs and contained candidate transcription factors including YABBY and WRKY. Population genomics revealed that despite a relatively homogeneous genetic background, localized selective sweeps on chromosomes 3 and 6 were associated with salt tolerance. By integrating transcriptomic interaction networks with population evolutionary signatures, five core candidate genes involved in abscisic acid biosynthesis and redox homeostasis were identified, highlighting a probable genetic basis for salt tolerance.

**Discussion:**

In summary, these findings suggest a dual-layered mechanism for foxtail millet salt tolerance, likely coordinated by localized adaptive selection at core genomic nodes and dynamic plasticity within transcriptional networks. This model offers insights into mitigating the growth-stress trade-off, presenting potential candidate targets for breeding resilient cereal crops.

## 1 Introduction

Soil salinization is one of the most pervasive abiotic stresses limiting crop productivity worldwide (Singh, 2022). Excessive salt induces both ionic toxicity and osmotic challenges on plants, leading to growth inhibition, metabolic dysfunction, membrane injury, and ultimately severe yield reduction. The interplay among osmotic stress, ionic imbalance, oxidative damage, and hormonal signaling leads to plant injury or even death under salinity. Thus, elucidating the molecular mechanisms underlying plant salt tolerance is essential for improving crop resilience and securing global food supply.

Foxtail millet (*Setaria italica* (L.)) is an emerging model of C_4_ crop with drought and stress tolerance abilities. It is of small genome (~510 Mb) with abundant genetic resources, and a short life cycle (Bennetzen et al., 2012). There are rich foxtail millet germplasm resources in China, accounting for over 70% of the global total. In recent years, significant advances have been made in foxtail millet genomics research. Jia et al. (2013) resequenced 916 accessions to construct the first haplotype map; Li et al. (2021) performed deep resequencing of 312 core accessions; He et al. (2023) constructed the first graph-based pan-genome from 1,844 *Setaria* accessions; and a complete T2T reference genome with the *Setaria*-db database has recently been released (He et al., 2024). Foxtail millet is becoming an important model for deciphering abiotic stress responses in C_4_ crops based on these achievements.

Salt tolerance in foxtail millet is a complex polygenic trait driven by intricate molecular networks. Early studies evaluating 155 core accessions revealed abundant phenotypic variation for salt tolerance (Krishnamurthy et al., 2014); concurrently, 159 differentially expressed genes were identified between contrasting cultivars using suppression subtractive hybridization (Puranik et al., 2011). As sequencing technologies have advanced, multi-omics approaches have provided a more comprehensive perspective for dissecting these stress responses. For instance, the integration of transcriptomic, metabolomic, and phosphoproteomic data has uncovered essential metabolic pathways and phosphorylation-mediated signaling cascades in foxtail millet (Pan et al., 2020, 2021). Furthermore, several key genes, such as *SiGRF1*, *SiMYB16*, and *SiGSTU24*, have been functionally characterized (Liu et al., 2020; Yu et al., 2023; Zhang et al., 2025). Building upon this foundation, recent research utilizing time-series RNA-seq coupled with Weighted Gene Co-expression Network Analysis (WGCNA) further proposed that salt tolerance likely arises from coordinated regulatory networks coupling ion homeostasis, stress signaling, and metabolic reprogramming, rather than solely from isolated single-gene effects (Liu et al., 2026).

Despite these advances in functional genomics, the evolutionary drivers shaping salt tolerance divergence at the population level remain underexplored in foxtail millet. Selective sweep analysis offers a robust method to identify genomic regions subjected to environmental directional selection. Selective sweep analysis has proven highly effective for screening salt tolerance genes in various crops. For instance, recent studies have successfully scanned genome-wide selection signatures and domestication patterns among 3,010 Asian cultivated rice accessions (Wang et al., 2018), and pinpointed the specific evolutionary signals underlying the *GATA19*-*SAT1/OsCYL4* saline-alkali tolerance module (Li et al., 2025). Despite these successful applications, its utilization in foxtail millet remains limited. More importantly, a critical question remains unresolved: is salt tolerance divergence primarily driven by adaptive evolution in coding sequences, stress-induced regulatory network reprogramming, or a synergistic interaction of both? To better understand this process, integrating genotype-by-environment interaction analysis and multiWGCNA with genome-wide selective sweep scanning may help systematically elucidate the genetic architecture of salt tolerance from the dual perspectives of sequence variation and regulatory plasticity.

Based on this background, we constructed a panel comprising 18 extreme salt-tolerant (STP) and 18 extreme salt-sensitive (SSP) accessions from a previously characterized germplasm pool. These two groups share similar genetic backgrounds, which effectively minimizes the confounding effects of population structure. An integrated dataset encompassing phenomics, physiology, transcriptomics, and population genomics was utilized for three main objectives: first, to dissect the differences in transcriptomic responses between STP and SSP accessions under salt stress, with a particular focus on identifying genotype-by-environment interaction genes; second, to construct salt tolerance-specific co-expression modules and map their upstream transcription factor regulatory networks; and third, to elucidate the genetic driving forces underlying salt tolerance divergence through the cross-integration of selective sweep signals and transcriptomic dynamics. This study establishes a dual evolutionary strategy framework in foxtail millet, wherein sequence-level coding variants and dynamic regulatory network reprogramming function as complementary drivers of salt tolerance. These findings offer novel insights into the genetic architecture of crop abiotic stress adaptation and provide promising candidate targets for molecular breeding.

## 2 Materials and methods

### 2.1 Plant materials, growth conditions and treatments

Approximately 500 different foxtail millet germplasm accessions (prefixed with “BJ”) and Shandong local germplasm resources (prefixed with “SDD” and “SDP”) were used in this study. The seeds were sown in standardized 96-well seedling trays (40 × 60 × 15 cm) and grown using a hydroponic system. They were placed in a plant growth chamber under controlled conditions (temperature 22°C, light intensity 300–400 μmol m^-2^ s^-1^, 10 h light/14 h dark) for two weeks until the three-leaf seedling stage. All samples used in this study were obtained under these culture conditions. Then, salt treatments with 150 and 300 mmol/L NaCl were separately applied for salt tolerance and sensitivity screening, establishing a gradient stress to effectively distinguish sensitive lines at 150 mmol/L and isolate extreme tolerant accessions under 300 mmol/L. Phenotypic observations and photographs were recorded on the third and fifth days after NaCl treatment. Subsequently, 18 accessions with extreme phenotypes were selected from both the salt-tolerant and salt-sensitive panels for further analysis.

### 2.2 Histochemical detection of ROS in STP and SSP of foxtail millet

Salt-tolerant and salt-sensitive foxtail millet seedlings were cultivated under growth conditions described above. At the three-leaf stage, uniformly developed seedlings were treated with 300 mmol/L NaCl, while those treated with water served as the control.

For the detection of hydrogen peroxide (H_2_O_2_), 3,3’-diaminobenzidine (DAB) staining was performed using a DAB colorimetric kit (Solarbio, Beijing, China; Cat. No. DA1010) according to the manufacturer’s instructions. After two days of salt treatment, seedling leaves were collected and incubated in the DAB staining solution in the dark for 4 h. The reaction was subsequently terminated by washing the leaves 2–3 times with distilled water, and the samples were immediately observed and photographed.

For the detection of superoxide anions (O_2_^-^), nitro blue tetrazolium (NBT) staining was performed using NBT staining kit (Coolaber, Beijing, China; Cat. No. SK2030) following the manufacturer’s protocol. Seedling leaves were incubated in NBT staining solution in the dark for 48 h. After staining, the leaves were decolorized with 75% (v/v) ethanol until the chlorophyll background was fully removed, followed by observation and photography. The relative ROS accumulation and salt tolerance of different foxtail millet accessions were visually assessed by comparing staining intensity and pattern between salt-treated and control seedlings.

### 2.3 Measurement of relative electrical conductivity and relative chlorophyll content

To evaluate photosynthetic capacity and cell membrane stability, relative electrical conductivity (REC) and relative chlorophyll content (RCC) were measured in uniform foxtail millet seedlings at the three-leaf stage.

For REC measurements, aboveground tissues were sampled from seedlings at 0, 2, and 4 days after 300 mM NaCl treatment, with three biological replicates per time point. The REC was measured according to the method described by Lutts et al. (1996) with minor modifications. Briefly, leaf segments were immersed in deionized water, and the initial electrical conductivity (R_1_) was measured after incubation. The samples were then boiled to kill the tissues completely, cooled to room temperature, and the final conductivity (R_2_) was recorded. REC (%) was calculated as (R_1_/R_2_) × 100%.

Concurrently, the RCC was measured using a portable chlorophyll meter (YLS-A; Zhejiang Top Instrument Co., Ltd., Hangzhou, China). Ten leaves were measured per variety to ensure data reliability and representativeness, and the values were averaged for comparative analysis.

### 2.4 Library construction and transcriptome sequencing

To investigate the transcriptional response to salt stress, leaf tissues were harvested from 36 foxtail millet accessions (18 salt-tolerant and 18 salt-sensitive; three biological replicates each) at the three-leaf stage following a 24 h treatment with or without 250 mM NaCl. Notably, a slightly lower concentration of 250 mM NaCl, rather than 300 mM, was selected for transcriptomic analysis as an optimal sub-lethal dose to avoid severe cellular mortality and ensure higher accuracy of the transcriptional data. Total RNA was extracted using FreeZol Reagent (R711, Vazyme, China), and its purity and integrity were verified via a NanoDrop 2000 spectrophotometer (Thermo Scientific, USA) and agarose gel electrophoresis. For library construction, poly(A) mRNA was enriched using oligo(dT) magnetic beads, enzymatically fragmented, and reverse-transcribed into double-stranded cDNA. Following end-repair, A-tailing, and MGI-adapter ligation, the cDNA fragments were PCR-amplified and purified. Ultimately, the libraries were circularized to generate DNA nanoballs (DNBs) and sequenced on the DNBSEQ platform (MGI Tech, China) according to the manufacturer’s instructions.

### 2.5 Differential expression analysis, functional enrichment, and interaction pattern classification

Differential expression analysis between groups was performed using the DESeq2 R package (Love et al., 2014). By constructing a design matrix, four statistical comparisons were established. The thresholds for identifying differentially expressed genes (DEGs) were set to a Benjamini-Hochberg (Benjamini and Hochberg, 1995) adjusted *P*-value (*P*adj) < 0.05 and |log_2_ (Fold Change)| > 1.

Gene Ontology (GO) and Kyoto Encyclopedia of Genes and Genomes (KEGG) pathway enrichment analyses for the DEGs were conducted using clusterProfiler (v4.0) (Wu et al., 2021). These analyses utilized all 28,790 expressed genes in the genome as the background set, calculated significance via hypergeometric tests, and applied the Benjamini-Hochberg (Benjamini and Hochberg, 1995) method for multiple testing correction (*P*adj < 0.05).

DEGs exhibiting significant interaction effects were classified into four primary response patterns based on the direction and statistical significance of their log_2_ (Fold Change) in both the salt-tolerant and salt-sensitive panels. Following this classification, GO and KEGG pathway enrichment analyses were performed separately for each response pattern.

### 2.6 Weighted gene co-expression network analysis

Independent gene co-expression networks were constructed for the salt-tolerant (n = 18) and salt-sensitive (n = 18) panels utilizing the multiWGCNA R package (Tommasini and Fogel, 2023). The network type was set to signed hybrid, and the optimal soft-thresholding power was determined based on the scale-free topology fit criterion. To assess the module preservation of the salt-tolerant network within the salt-sensitive network, the modulePreservation function was employed (Langfelder et al., 2011), where a *Z*_summary_ score < 2 was defined as an unpreserved module. Module eigengenes (MEs) were further calculated, and differential module eigengene (DME) analysis was conducted using either a paired Student’s *t*-test or a Wilcoxon signed-rank test, depending on data normality, to evaluate the dynamic changes in module expression levels pre- and post-salt treatment.

### 2.7 Transcription factor annotation and transcription factor binding site enrichment analysis

Transcription factor (TF) families of the module genes were predicted and annotated utilizing the PlantTFDB v5 database (Tian et al., 2020). Promoter regions, defined as the 2-kb sequence upstream of the transcription start site (TSS), were extracted for all genes across the genome. Subsequently, the FIMO tool from the MEME Suite (v5.5.9) (Bailey et al., 2009) was used to scan these promoter sequences against the non-redundant plant motif database from JASPAR2024 CORE (Rauluseviciute et al., 2024) to identify potential TFBSs, with a match threshold set at *P* < 1×10^-4^. Using the genome-wide promoter regions as the background, hypergeometric tests were performed to identify significantly enriched TFBSs within the promoters of the module genes. The significance criteria were defined as a false discovery rate (FDR) < 0.05 and a fold enrichment > 1.

### 2.8 Library construction, genome resequencing, and population structure analysis of STP and SSP

At the three-leaf stage, uniform foxtail millet seedlings were subjected to 250 mM NaCl treatment for 24 h, whereas the control plants were maintained under non-stressed conditions. Total genomic DNA was isolated from the entire seedlings utilizing the cetyltrimethylammonium bromide (CTAB) method. For sequencing library construction, the extracted DNA underwent enzymatic fragmentation and adapter ligation, followed by magnetic bead-based size selection (300–700 bp) and purification. Whole-genome resequencing was subsequently conducted on the DNBSEQ-T7 platform (MGI, Shenzhen, China). The generated sequencing reads were mapped to the reference genome using the Burrows-Wheeler Aligner (BWA) (Li, 2013) to facilitate single nucleotide polymorphism (SNP) calling. Finally, the resulting variant dataset was employed for population structure profiling and the detection of selective sweeps.

To elucidate the genetic relationships between the two foxtail millet panels, population structure was inferred using ADMIXTURE v1.3.0 (Alexander et al., 2009), and principal component analysis (PCA) was conducted employing GCTA v1.94.1 (Yang et al., 2011). The resulting population structure and PCA plots were subsequently visualized using the R package ggplot2 v3.4.2 (Villanueva and Chen, 2019). To identify genomic regions under selection (selective sweeps), a comprehensive whole-genome scan was performed by integrating the genetic differentiation index (*F_ST_*) and the cross-population composite likelihood ratio (XP-CLR) approaches. Specifically, pairwise *F_ST_* values between the populations were calculated using VCFtools v0.1.16 (Danecek et al., 2011). Concurrently, the XP-CLR method (Chen et al., 2010) was utilized to detect selection signatures based on a probability model of multilocus allele frequency divergence between the populations. Both *F_ST_* and XP-CLR statistics were computed across each chromosome utilizing a unified sliding window strategy, with the parameters uniformly set to a window size of 20 kb and a step size of 10 kb.

## 3 Results

### 3.1 Physiological differentiation between STP and SSP of foxtail millet

Based on a combination of field experiments and laboratory screening of over 500 foxtail millet accessions, 18 highly salt-tolerant and 18 extremely salt-sensitive lines were selected to construct a salt-tolerant panel (STP) and a salt-sensitive panel (SSP), respectively. The STP and SSP exhibited marked phenotypic divergence (**Figure 1**; **Supplementary Figure 1**). Foxtail millet seedlings at the three-leaf stage were subjected to salt stress under hydroponic conditions, and their phenotypic responses were recorded at 3 and 5 days post-treatment. Under 300 mM NaCl treatment, the STP seedlings maintained green and relatively upright leaves, sustained steady growth, and exhibited robust overall vigor after 3 days of treatment, with chlorosis and wilting symptoms delayed until after 5 days of treatment (**Figure 1A**). In contrast, the SSP seedlings displayed severe salt-induced damage after 3 days of treatment, characterized by pronounced leaf chlorosis and wilting, and the majority of the plants had died by the fifth day of treatment (**Figure 1B**). Similarly, under the 150 mM NaCl treatment, the STP showed no significant phenotypic changes relative to the control (**Supplementary Figure 1A**), whereas the SSP exhibited severe leaf chlorosis and wilting after both 3 and 5 days of treatment (**Supplementary Figure 1B**).

**Figure 1:**
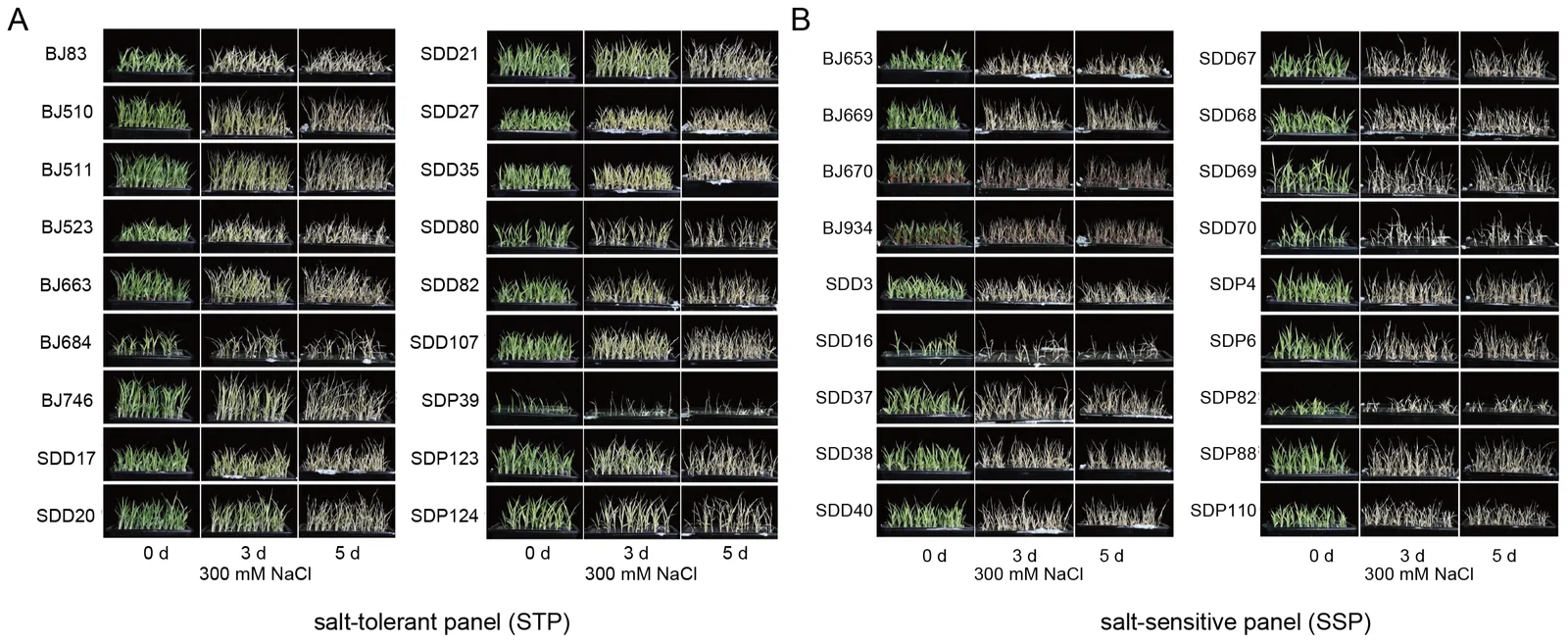
Phenotypic responses of the salt-tolerant panel (STP) and salt-sensitive panel (SSP) in foxtail millet under 300 mM NaCl treatment. **(A)** Salt-tolerant panel (STP) **(B)** Salt-sensitive panel (SSP). Representative images illustrating phenotypic differences among various foxtail millet lines subjected to salt stress. Accession names are indicated on the left. From left to right, the photographs display plant phenotypes at 0 (control), 3, and 5 days post-treatment.

### 3.2 Histological staining of seedling leaves from the STP and SSP of foxtail millet

The antioxidant capacity of STP and SSP was assessed by histological staining. Under normal growth conditions, there was almost no significant difference in H_2_O_2_ content between the two panels, as evidenced by their similar DAB staining patterns (**Figure 2A**). However, after salt stress treatment, STP exhibited lighter reddish - brown staining in the leaves, indicating lower H_2_O_2_ content compared with those of SSP (**Figure 2B**). This suggests that STP have a stronger ability to scavenge peroxides under salinity. In contrast, some SSP showed deeper staining, mostly dark brown, in the leaves, indicating more H_2_O_2_ accumulation and weaker ability to scavenge peroxides in the seedlings under salinity, which implies poorer salt tolerance abilities (**Figure 2B**).

**Figure 2:**
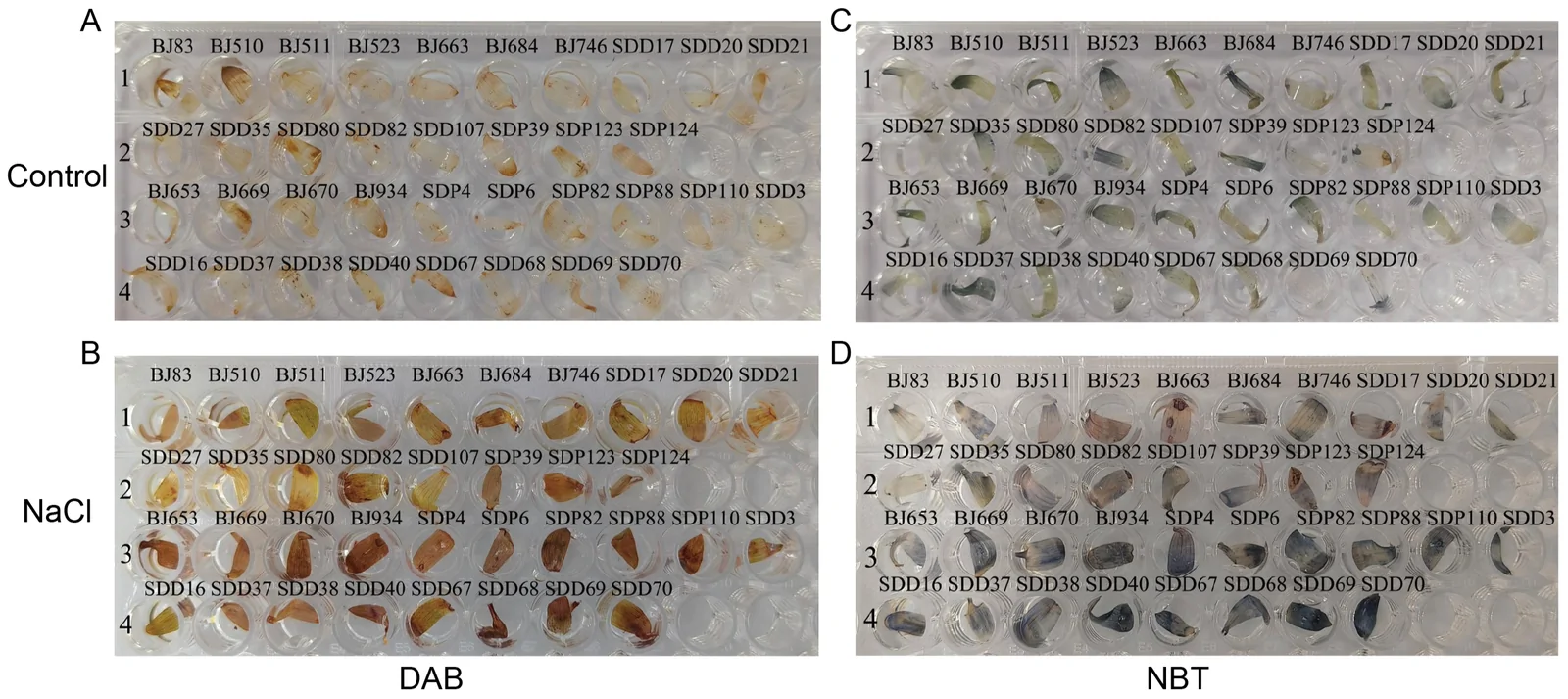
Histological staining of seedling leaves of STP and SSP. **(A)** DAB staining of seedling leaves from the control group of STP and SSP. **(B)** DAB staining of seedling leaves under 300 mM NaCl treatment. **(C)** NBT staining of seedling leaves from the control group of STP and SSP. **(D)** NBT staining of seedling leaves under 300 mM NaCl treatment. In each panel, the 1st and 2nd rows present the STP leaves, while the 3rd and 4th rows show the SSP leaves.

Similarly, NBT staining was performed to detect superoxide anion (O_2_^−^) accumulation. Under normal conditions, the staining intensities were comparable between the STP and SSP (**Figure 2C**). Under salt stress, however, the STP displayed a lighter blue color than the SSP, indicative of reduced O_2_^−^ accumulation (**Figure 2D**). This suggests that the STP has a stronger capacity to scavenge superoxide anions under salt stress. Conversely, the SSP exhibited intense dark blue staining in the leaves, demonstrating elevated O_2_^−^ levels and a weaker ROS-scavenging ability under salinity (**Figure 2D**).

### 3.3 Physiological responses between STP and SSP under salt stress

To evaluate the extent of leaf damage, we measured the relative chlorophyll content (RCC) of each foxtail millet line within the STP and SSP, using 10 seedlings per line. These measurements were taken before salt treatment, as well as after 2 and 4 days of treatment. The mean RCC for each panel was subsequently calculated. The results showed that before salt treatment, STP and SSP exhibited comparable baseline RCC values with no significant differences (**Figure 3A**). However, after 2 days of treatment, the mean RCC of the STP was 5.9, which was significantly higher than that of the SSP. After 4 days of treatment, although the RCC of the STP had slightly decreased to 3.7, it remained significantly higher than the 1.6 observed in the SSP (**Figure 3A**). These results suggest that STP can better maintain chlorophyll levels under salt stress, thereby helping to mitigate salt-induced damage to the plants.

**Figure 3:**
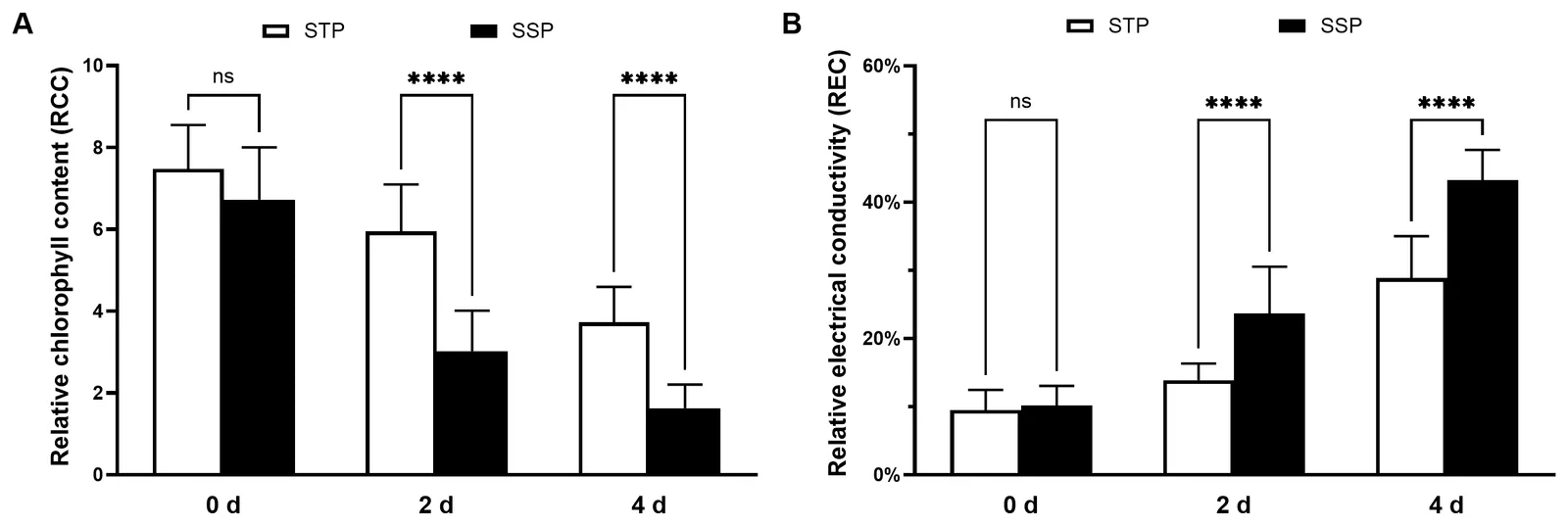
Physiological responses between STP and SSP under salt stress. **(A)** Relative chlorophyll content (RCC) before and after salt treatment. **** represents a significant difference at the *P* < 0.0001 level (*n* = 10). **(B)** Relative electrical conductivity (REC) before and after salt treatment. **** represents a significant difference at the *P* < 0.0001 level (*n* = 3).

To assess cell membrane integrity, the relative electrical conductivity (REC) of each line was measured and calculated before and after salt treatment, and the mean REC values for the STP and SSP were determined (**Figure 3B**). Prior to salt treatment, the initial REC values of the STP and SSP were similar, indicating comparable membrane integrity under non-stress conditions. After 2 days of treatment, the REC of the SSP increased to 24%, which was significantly higher than the 14% observed in the STP, indicating more severe membrane damage in the salt-sensitive panel. After 4 days of treatment, the REC of the SSP further elevated to 43%, substantially higher than the 29% recorded for the STP (**Figure 3B**). Although the REC of the STP also increased, the magnitude of this increment was markedly smaller than that of the SSP. This further implies that STP possesses greater salt tolerance and an enhanced capacity to maintain membrane integrity under saline conditions.

### 3.4 Transcriptional divergence and regulatory network analysis of STP and SSP

To investigate the underlying mechanisms driving salt tolerance divergence between the two foxtail millet panels, transcriptome sequencing (RNA-seq) was performed. Expression data were obtained from 72 samples, identifying 28,784 expressed genes for differential expression analysis (**Figure 4A**). Four comparison groups were established: Tolerant (STP Salt vs. STP Control) and Sensitive (SSP Salt vs. SSP Control) to evaluate gene expression responses before and after salt stress; Baseline (SSP Control vs. STP Control) to assess constitutive expression differences under normal conditions; and Interaction (Tolerant vs. Sensitive) to identify genes with significant variance in salt response amplitude between the two panels (**Figures 4A, B**). The transcriptomic analysis identified 2,365 genes exhibiting a significant genotype-by-treatment interaction. These genes displayed distinct salt-responsive patterns between the two groups and serve as key candidates dictating salt tolerance divergence (**Figure 4A**).

**Figure 4:**
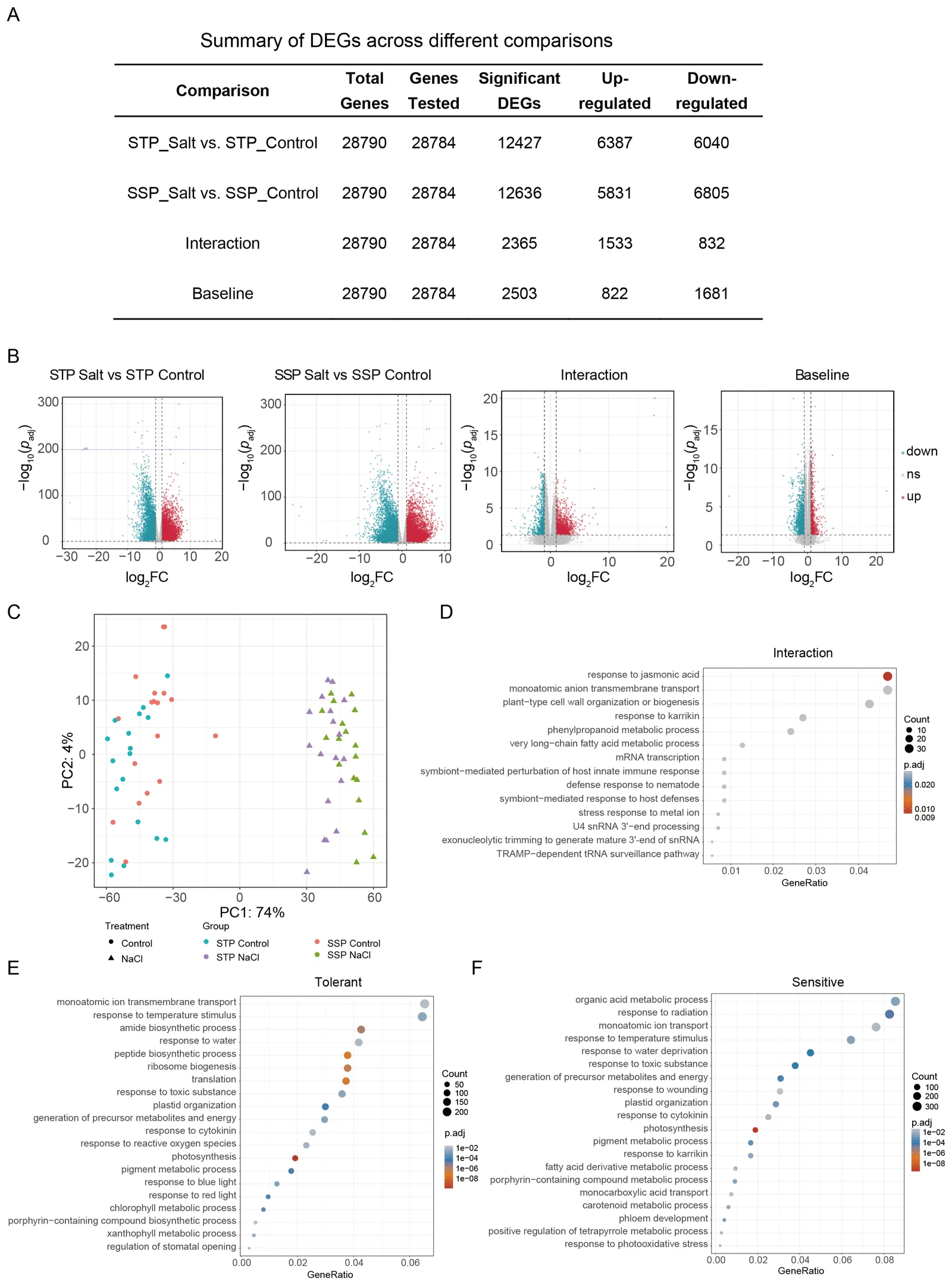
Transcriptome profiling and differentially expressed genes (DEGs) analysis. **(A)** DEGs of different comparison groups. **(B)** Volcano plots of DEGs in corresponding comparison groups. **(C)** Principal component analysis (PCA) of all transcriptome samples. **(D–F)** Bubble plots of significant enrichment analysis for DEGs. The major biological processes involved in the Interaction **(D)**, Tolerant **(E)**, and Sensitive **(F)** groups are shown, respectively.

Principal component analysis (PCA) revealed that salt treatment was the primary driver of transcriptional variation (PC1), followed by genotype (**Figure 4C**). A sample distance heat-map further confirmed the significant impact of salt stress and genotype on transcriptomic profiles (**Supplementary Figure 2**).

Gene Ontology (GO) and Kyoto Encyclopedia of Genes and Genomes (KEGG) enrichment analyses were conducted for the differentially expressed genes (DEGs) (**Figures 4D–F**; **Supplementary Figure 3**). In the tolerant panel, salt-responsive DEGs were primarily enriched in broad-spectrum abiotic stress responses, including transmembrane ion transport, photosystem remodeling, and oxidative stress defense (**Figure 4E**), and were significantly mapped to KEGG pathways related to photosynthesis, energy metabolism, and global metabolic networks (**Supplementary Figure 3A**). Conversely, DEGs in the sensitive panel were inclined toward physiological restructuring, such as osmotic regulation, plastid organization, and fatty acid derivative synthesis (**Figure 4F**), sharing similar primary KEGG pathways with the tolerant group (**Supplementary Figure 3B**). Notably, DEGs with significant genotype-environment interactions were specifically enriched in jasmonic acid-mediated defense responses, phenylpropanoid secondary metabolism, and post-transcriptional RNA processing (**Figure 4D**). This was corroborated by KEGG analysis, which highlighted significant enrichment in the phenylpropanoid biosynthesis pathway (**Supplementary Figure 3C**). These findings suggest that the reprogramming of complex secondary metabolic networks and transcriptional regulatory divergence constitute the core molecular basis for the differential salt tolerance observed between the two panels.

### 3.5 Expression pattern clustering and functional enrichment of salt tolerance candidate genes

To further delineate core candidate genes associated with salt tolerance, the expression patterns of genes in the interaction group were subdivided into five categories (I, II, III, IV, and V), comprising 594, 718, 122, 907, and 24 genes, respectively (**Figure 5A**). Among these, the 594 genes in Category I exhibited specific responsiveness in the tolerant panel, correlating highly with the salt-tolerant phenotype, and were thus identified as the pivotal candidate gene cluster determining the tolerance mechanism. GO enrichment analysis was performed to elucidate the specific biological functions of these distinct expression patterns under salt stress, revealing highly divergent yet complementary adaptive networks among the groups. Category I genes dominated the transcriptional response, clustering into two highly interconnected core functional modules (**Figure 5B**). The first module governed extensive post-transcriptional RNA regulation, showing extreme enrichment in nuclear RNA surveillance, snRNA processing, RNA degradation, and mRNA export, establishing post-transcriptional quality control as a critical stress-response node. The second module focused on direct physiological adaptation, specifically stomatal movement and closure mediated by lipid phosphorylation pathways. This indicates that complex post-transcriptional regulation combined with lipid-signaling-driven stomatal defense constitutes the primary salt tolerance mechanism in Category I genes (**Figure 5B**). The remaining categories provided peripheral complementary roles in developmental delay, basal metabolism, and structural remodeling. Specifically, Category II genes coordinated morphological development of aerial parts and reproductive organs, jasmonic acid/salicylic acid-mediated cross-defense responses, and the construction of lipid physical barriers such as suberin (**Supplementary Figure 4**). Category III genes predominantly regulated macromolecular synthesis and physiological network remodeling, including ribosome biogenesis and protein translation (**Supplementary Figure 5**). Category IV genes responded directly to environmental stress, primarily encompassing anion-dominated transmembrane ion transport, anti-dehydration barrier reconstruction driven by cell wall and wax metabolism, and multiple endogenous hormone signaling pathways involving auxin and brassinosteroids (**Supplementary Figure 6**).

**Figure 5:**
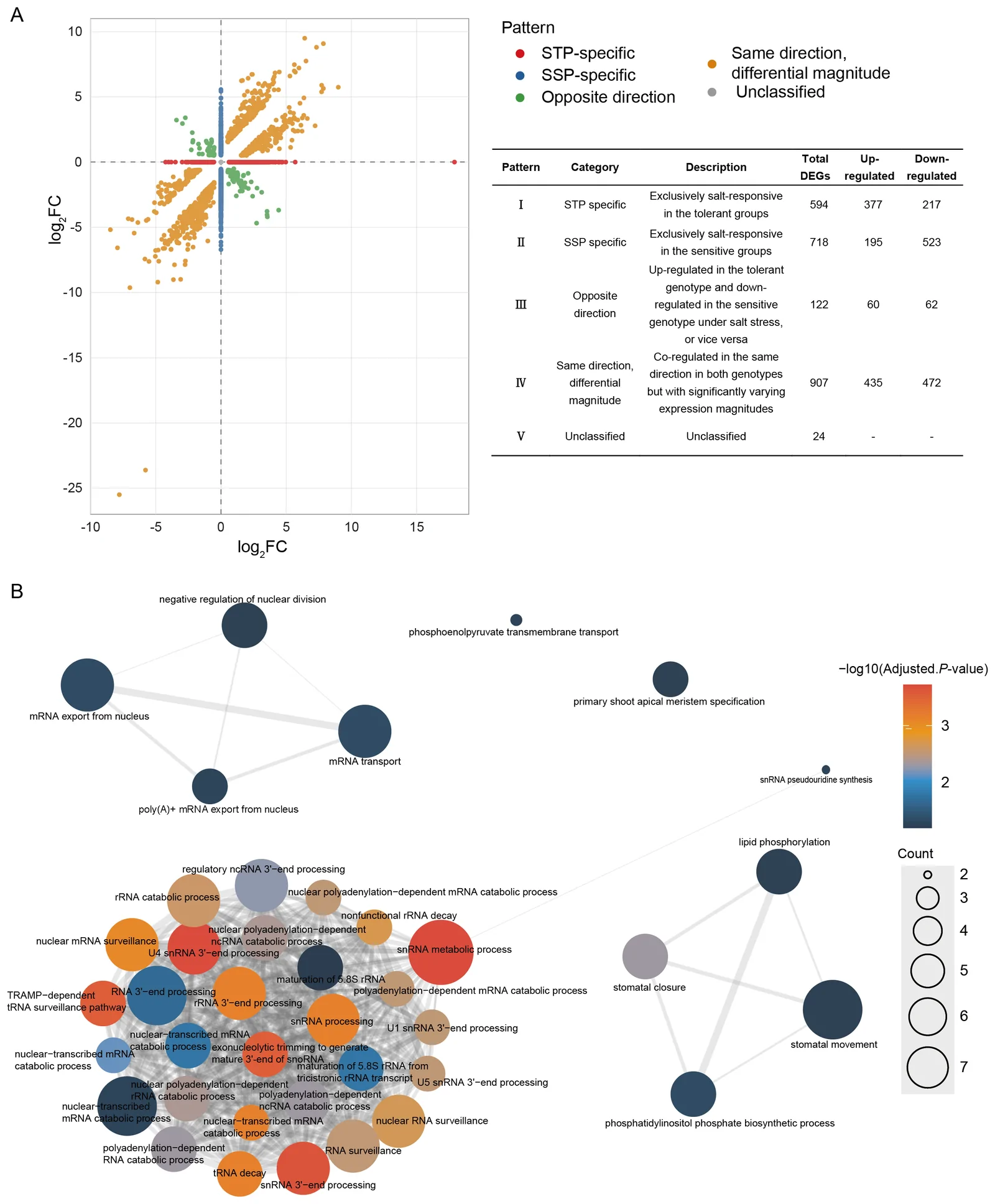
DEGs analysis with genotype-by-treatment interaction under salt stress. **(A)** Scatter plot of expression patterns and statistical classification of interaction DEGs. The right panel summarizes the classification criteria and the total number of up- and down-regulated genes per pattern. **(B)** Enrichment Map of significant GO biological processes for interaction DEGs. Nodes represent significantly enriched GO terms, and connecting lines indicate shared genes between different pathways.

### 3.6 WGCNA reveals panel-specific co-expression modules and regulatory networks underlying salt tolerance divergence

To elucidate panel-specific and conserved regulatory patterns, WGCNA was applied, and 10 distinct modules in both the STP and SSP were identified, respectively. Module preservation analysis indicated that 19 of the 20 total modules were preserved across networks (Z*summary* > 2). Notably, only the STP007 module (including 81 genes) was unpreserved (Z*summary* = 1.34), thus functioning as a salt-tolerant-specific co-expression regulatory unit (**Figures 6A, B**).

**Figure 6:**
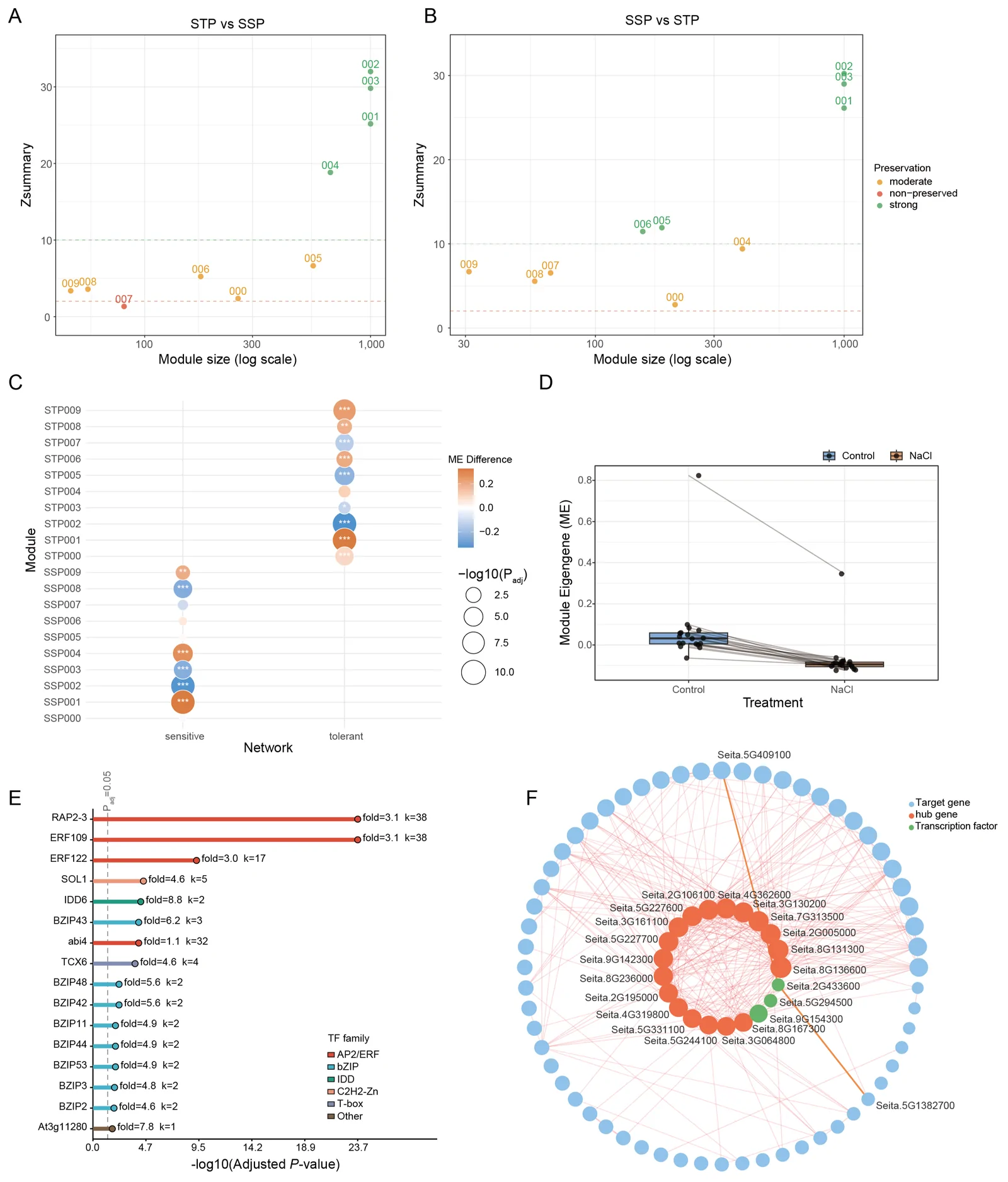
WGCNA and identification of core modules and key genes. **(A, B)** Module preservation analysis. Preservation scores of modules in the counterpart genotype dataset are shown using STP **(A)** and SSP **(B)** as reference networks. **(C)** Differential analysis of ME. **(D)** Boxplots of candidate core ME expression patterns under control and NaCl treatments. Scatter points and connecting lines represent ME trajectories of independent samples. **(E)** Bar chart of significantly enriched TF families within the core module. **(F)** Visualization of the co-expression network of key genes within the core module. Orange nodes represent core hub genes, green nodes denote transcription factors, and light blue peripheral nodes indicate co-expressed target genes. Connecting lines represent expression correlations.

Analysis of module eigengenes (MEs) revealed that, aside from SSP005, SSP006, SSP007, and STP004, the majority of modules exhibited significant transcriptional reprogramming in response to salt stress. Interestingly, the eigengene trajectory of the salt-tolerant-specific STP007 module showed a marked down-regulation post-stress (**Figures 6C, D**). Subsequent functional annotation of the 81 genes comprising STP007 highlighted three critical transcription factors (TFs): a YABBY TF (*Seita.9G154300*) increasingly implicated in plant stress adaptation, alongside two well-characterized stress-responsive WRKY TFs (*Seita.5G294500* and *Seita.2G433600*).

Analysis of transcription factor binding sites (TFBSs) across the promoter regions of this module’s genes revealed 17 significantly enriched motifs, clustering into five major classes. Notably, AP2/ERF family motifs were associated with the largest subset of target genes, implicating ethylene or abscisic acid (ABA) signaling pathways as primary regulators of this module. Additionally, the IDD family showed the highest fold enrichment, underscoring a strong potential regulatory role for C2H2-ZF transcription factors (**Figure 6E**).

Construction of a hierarchical regulatory network from the module’s constituents revealed a cascading amplification of transcriptional signals (**Figure 6F**). A central regulatory core- comprising the three aforementioned TFs and 18 highly connected hub genes coordinated the expression of downstream targets. Notably, the central WRKY TFs showed strong co-expression correlations with *Seita.5G382700* and *Seita.5G409100*. The identification of WRKY cis-regulatory elements within their respective promoters provides compelling evidence for direct transcriptional regulation (**Figure 6E**).

### 3.7 Genetic differentiation and panel structures of STP and SSP

To investigate the genetic background and assess the degree of differentiation between the two foxtail millet groups, the panel structures of the STP and SSP were analyzed. High-depth whole-genome resequencing was performed, yielding an average of 114,979,080 raw reads per individual. The resulting sequences were aligned to the Yugu1 T2T reference genome, achieving a high average mapping rate of 90.44% (ranging from 81.94% to 98.33%) and an average sequencing depth ranging from 29.28× to 47.95×. Initially, 8,097,738 variants were identified. Following rigorous filtering, 5,087,901 high-quality single nucleotide polymorphisms (SNPs) with a missing rate of less than 20% were retained. The subsequent exclusion of SNPs with a minor allele frequency (MAF) of less than 0.05 yielded a final dataset of 2,501,642 high-confidence SNPs.

Based on a subset of 126,074 unlinked SNPs, the panel structure analysis aligned with expectations. At *K* = 2, the STP and SSP did not separate into two distinct clusters (**Figure 7A**). Consistently, PCA did not reveal significant differentiation between the two panels (**Figure 7B**). Collectively, these results demonstrated the absence of a pronounced structure dividing of the STP and SSP.

**Figure 7:**
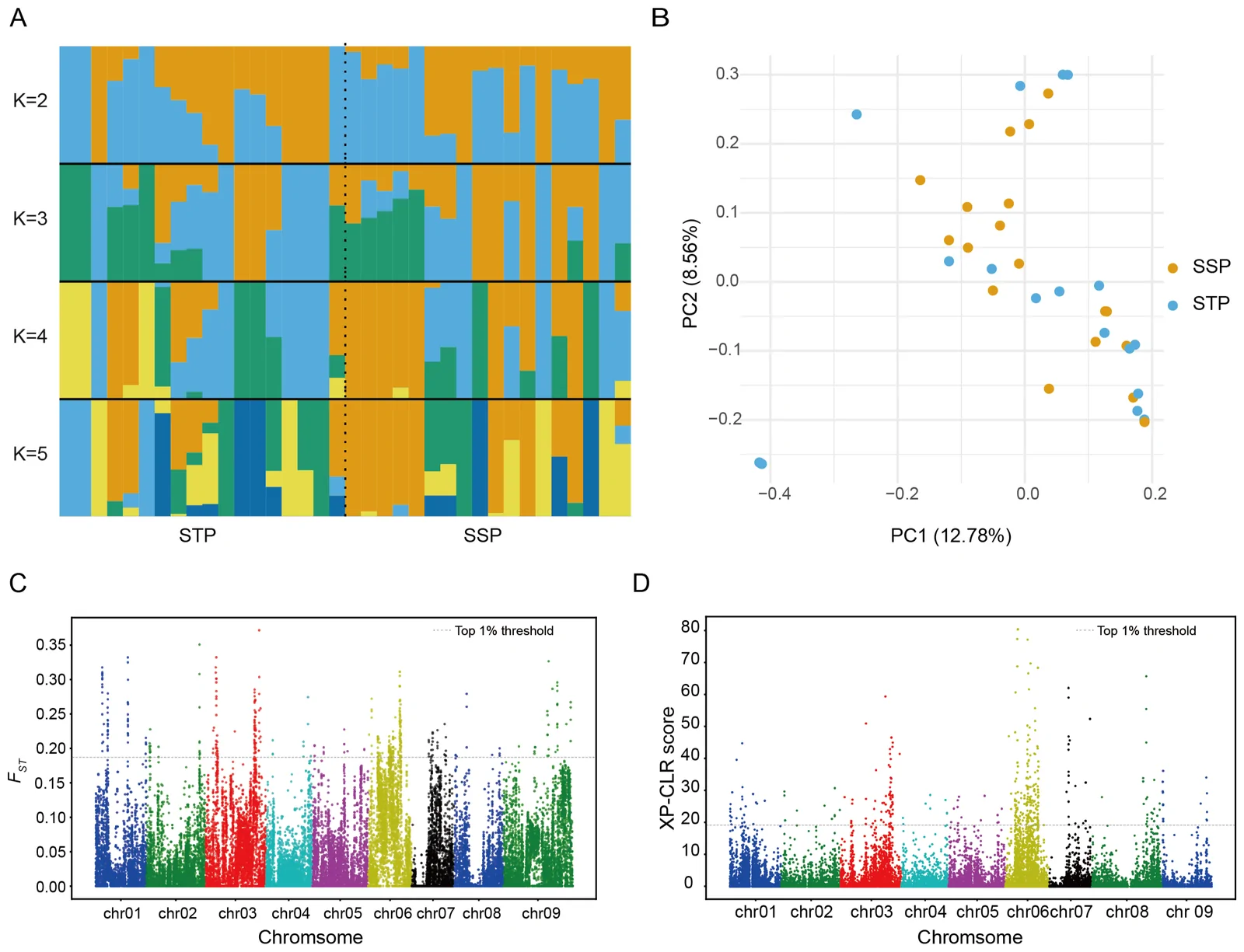
Panel structure and selective sweep analysis between STP and SSP of foxtail millet. **(A)** STRUCTURE analysis of STP and SSP (*K* = 2-5). Each vertical bar represents a single accession, with colors indicating estimated ancestry membership coefficients. **(B)** Principal component analysis (PCA). Blue and orange dots correspond to accessions from the STP and SSP groups, respectively. **(C)** Genome-wide selection sweep scan based on the fixation index (*F*_ST_). The horizontal dashed line indicates the genome-wide significance threshold at the top 1% level. **(D)** Genome-wide selection sweep scan based on XP-CLR scores. The horizontal dashed line represents the genome-wide significance threshold at the top 1% level.

Despite this highly homogeneous genetic background, locus-specific selective sweeps driven by salt tolerance were discernible. Genome-wide scans were conducted using the fixation index (*F*_ST_) and cross-population composite likelihood ratio (XP-CLR) to identify these regions. At a stringent top 1% threshold, both metrics consistently identified prominent selection signals on chromosomes 3 and 6, characterized by highly synchronized *F_ST_* elevations (**Figure 7C**) and sharp XP-CLR peaks (**Figure 7D**). Overlapping candidate regions with moderate intensities were also detected on chromosomes 07 and 09. The high congruence between these independent statistical approaches strongly indicates that these localized genomic regions harbor key functional genes modulating salt tolerance divergence in foxtail millet.

### 3.8 Cross-validation and functional integration of candidate genes

To decipher the genetic basis of salt tolerance in foxtail millet, we integrated transcriptomic interaction networks with population evolutionary signatures by cross-referencing genes from the four previously identified key responsive modules with genome-wide selective sweep signals (*F_ST_* and XP-CLR, using a stringent top 1% threshold). This analysis pinpointed five crucial determinants under strong diversifying selection, which are broadly involved in core pathways such as hormone regulation, redox homeostasis, and stress signal transduction. Specifically, Module I contains three genes: *Seita.6G138300*, an ortholog of *Arabidopsis* CNX2, which directly influences plant salt and drought tolerance by regulating ABA biosynthesis (Schwarz and Mendel, 2006; Bittner, 2014); *Seita.7G058300*, encodes a protein possessing the DUF26 (PF01657) domain, a defining hallmark of the cysteine-rich receptor-like kinase (CRK) family, whose members are well documented to participate in various abiotic stress responses, such as drought, salinity, and ABA signaling pathways (Zhang et al., 2023); and *Seita.9G392800*, which lacks homologous functional annotations and serves as a promising novel target for salt tolerance. Module IV harbors two genes: *Seita.2G037700*, which is highly homologous to the H-type thioredoxins in rice and Arabidopsis, has been demonstrated to enhance plant tolerance to abiotic stresses by regulating redox homeostasis (Kamoun et al., 2024; Zhao et al., 2026); and *Seita.6G149600*, which is predicted to encode a small peptide. In stark contrast to these core targets, an independent analysis of the 81 co-expressed genes within the STP007 module revealed a complete absence of overlapping selective sweep signals under the same rigorous threshold. Transcriptomic analysis of gene expression levels (TPM) further revealed distinct expression patterns for the five strongly selected candidate genes and the three core transcription factors within the STP007 module. Upon salt stress, all five candidate genes consistently exhibited up-regulation in the STP, whereas their transcriptional responses diverged in the SSP (two up-regulated and two down-regulated). In contrast, the associated transcription factors were broadly down-regulated across both panels (**Supplementary Figure 7A**).

To determine whether the salt-tolerance divergence identified by transcriptomic responses and selective sweep signatures possesses a sequence-level variation basis, we performed genotypic characterization and association analysis for these eight genes across the STP and SSP panels using whole-genome SNP data (2,501,642 SNPs, MAF ≥ 5%). Allele frequency comparisons via Fisher’s exact test demonstrated a significant enrichment of mutant alleles in the STP panel across all gene loci, with the sole exception of Seita.9G154300 (**Supplementary Figure 7B**). Furthermore, haplotype analysis indicated that the reference-like haplotypes of Seita.9G392800, Seita.7G058300, and Seita.6G149600 were significantly over-represented in the SSP panel. Conversely, a salt-tolerance-specific mutant haplotype of Seita.6G149600 was exclusively detected in the STP panel.

This striking divergence at the multi-omics intersection provides profound insights into the diversified genetic strategies of foxtail millet during its evolutionary adaptation to salt stress. On the one hand, phenotypic variation is driven by the sequence-level adaptive divergence of a few key node genes (e.g., targets in Modules I and IV). On the other hand, co-expression networks like STP007 circumvent drastic coding sequence mutations, relying primarily on high transcriptional synergy and regulatory plasticity to achieve flexible responses to salt stress.

## 4 Discussion

Salt stress severely impairs plant growth and limits yield by inducing osmotic stress, ion toxicity, and secondary oxidative stress (Zhu, 2016). Our results demonstrate that under salt conditions, the STP exhibits superior physiological resilience compared to the SSP. Specifically, the STP maintained a higher relative chlorophyll content and a lower relative electrical conductivity, reflecting an enhanced capacity to preserve the integrity of the photosynthetic apparatus and cell membrane stability. Furthermore, histochemical staining (DAB and NBT) confirmed that the STP utilizes a highly efficient ROS scavenging system to significantly attenuate the over-accumulation of H_2_O_2_ and O_2_^^-^^. Collectively, these findings suggest that sustaining intracellular redox homeostasis and structural integrity serves as a fundamental physiological defense mechanism against salt stress in foxtail millet, which aligns with previous studies on stress tolerance in cereal crops (Kopecká et al., 2023).

Transcriptomic analysis reveals that foxtail millet salt adaptation is likely driven by complex multigenic regulatory networks rather than constitutive expression differences. Interaction analysis identified 2,365 DEGs with significant genotype-by-treatment effects, indicating that phenotypic divergence is largely attributable to transcriptional response reprogramming. Category I genes which specifically responsive in STP, clustered into two core functional modules: one governing post-transcriptional quality control (such as nuclear RNA surveillance, snRNA processing, mRNA export) and another that may mediate lipid-phosphorylation driven stomatal closure. This synergistic activation of RNA regulatory networks and physical barrier responses appears to constitute a primary defense strategy of STP.

WGCNA further identified the STP007 module as a tolerance-specific co-expression unit featuring three hub transcription factors, two WRKYs (*Seita.5G294500*, *Seita.2G433600*) and one YABBY (*Seita.9G154300*), likely forming a regulatory cascade. WRKY TFs are classic regulators of plant abiotic stress responses, typically modulating target genes by binding to W-box cis-elements downstream of the ABA and MAPK signaling pathways (Jiang et al., 2017), while emerging evidence increasingly highlights pivotal roles of YABBY TFs in mediating environmental stress responses (Wang et al., 2024). The significant enrichment of AP2/ERF and IDD binding motifs within the promoter regions of this module suggests a comprehensive regulatory network dominated by ethylene and ABA signaling, likely involving extensive phytohormone crosstalk.

The homogeneous genetic background between STP and SSP, as evidenced by STRUCTURE (*K* = 2) and PCA analyses showing no significant panel stratification, reflects the shared domestication history of foxtail millet landraces. Despite genome-wide homogeneity, strong selective sweeps on chromosomes 3 and 6 indicate that salt-driven directional selection did not induce genome-wide divergence, but instead precisely targeted a few localized genomic regions. This pattern is consistent with a rapid evolutionary response where adaptive alleles rapidly rise in frequency within specific loci without requiring broad genomic differentiation (Nielsen, 2005). Cross-integration of these selective signals with transcriptomic modules identified five core genes under positive selection, including *Seita.6G138300* (CNX2 ortholog, ABA biosynthesis), *Seita.2G037700* (thioredoxin H-type, redox homeostasis), and *Seita.7G058300* (CRK homolog, stress perception), which provide genetically stable anchors for tolerance. In contrast, the STP007 module showed a complete absence of selective sweep signals, revealing a strategy of dynamic regulatory plasticity, whereby AP2/ERF and IDD driven transcriptional plasticity enables rapid, reversible stress responses without relying on coding variants.

To validate this genetic architecture, we extended our molecular evidence from transcriptomic profiles to DNA sequence variations. Core candidate genes exhibited specific transcriptional induction in STP, while their mutant alleles and unique haplotypes were significantly enriched in this group. This pattern demonstrates a synchronized sequence variation to transcriptional induction response. Conversely, the enrichment of the Seita.9G154300 mutant allele in SSP suggests a potential negative regulatory or loss-of-function mechanism. This multi-omics cross-validation corroborates positive selection on these loci, highlighting Seita.5G294500 and Seita.6G149600 as key functional candidates.

By integrating adaptive variants at core nodes with dynamic downstream network reprogramming, this dual-layered architecture provides a plausible evolutionary model that may help mitigate the trade-off between growth stability and stress flexibility. For molecular breeding, it offers a two-pronged strategy: leveraging core genes for marker-assisted selection or allele-specific editing and utilizing network architectures such as STP007 for dynamic promoter engineering. Ultimately, these multi-omics insights provide valuable targets and a theoretical framework for breeding stress-resilient cereal crops.

## 5 Conclusions

In summary, this study elucidates a dual-layered evolutionary and regulatory mechanism underlying salt tolerance in foxtail millet. This mechanism is coordinately driven by two distinct strategies: first, the directional selection and enrichment of adaptive alleles at core genomic nodes, which maintain baseline physiological homeostasis; and second, the high dynamic plasticity of downstream transcriptional regulatory networks, which mediates rapid and reversible responses to environmental stress. This dual-mechanism model may contribute to mitigating the evolutionary trade-off between plant growth and stress resilience. Concurrently, these findings provide promising candidate targets for the engineering and breeding of high-yielding, stable, and climate-resilient cereal crops.

## Data Availability

The transcriptome and resequencing data for the salt-tolerant and salt-sensitive panels utilized in this study have been deposited in the CNCB (China National Center for Bioinformation), project numbers PRJCA068034.
