# Supplementary material for: Transcriptomics and resequencing reveal dual evolutionary strategies for salt tolerance in foxtail millet (Setaria italica (L.))
Source: Front Plant Sci. 2026 Sep 16;17:1938267. doi: 10.3389/fpls.2026.1938267 (PMC13623612; doi:10.3389/fpls.2026.1938267)
Supplement: Supplementary file 1 [file SupplementaryFile1.docx]

**Supplymentary Figure 1 Phenotypic responses of the salt-tolerant panel (STP) and salt-sensitive panel (SSP) in foxtail millet under 150 mM NaCl treatment.**

(A) Salt-tolerant panel (STP) (B) Salt-sensitive panel (SSP). Representative images illustrating phenotypic differences among various foxtail millet lines subjected to salt stress. Accession names are indicated on the left. From left to right, the photographs display plant phenotypes at 0 (control), 3, and 5 days post-treatment.

**Supplymentary Figure 2 Hierarchical clustering heatmap of sample-to-sample distances.**

The heatmap displays Euclidean distances based on global gene expression profiles. The color scale represents sample dissimilarity, transitioning from blue (high similarity) to red (low similarity). Top and left dendrograms illustrate sample clustering.

**Supplymentary Figure 3 KEGG pathway enrichment analysis of DEGs.**

Bubble plots showing the top enriched KEGG pathways for (A) STP, (B) SSP, and (C) the Interaction effect. The x-axis represents the GeneRatio, and bubble size indicates the number of enriched genes (Count). The color gradient corresponds to the adjusted p-value (p.adj), indicating the statistical significance of pathway enrichment.

**Supplymentary Figure 4 Functional enrichment network analysis of Category Ⅱ genes.**

Network visualization of enriched GO terms. Nodes represent individual GO terms, with sizes proportional to the number of enriched genes (Count). Edges between nodes denote functional similarity, while the color gradient reflects the statistical significance, represented by -log10 (Adjusted P-value).

**Supplymentary Figure 5 Functional enrichment network analysis of Category Ⅲ genes.**

Network visualization of enriched GO terms. Nodes represent individual GO terms, with sizes proportional to the number of enriched genes (Count). Edges between nodes denote functional similarity, while the color gradient reflects the statistical significance, represented by -log10 (Adjusted P-value).

**Supplymentary Figure 6 Functional enrichment network analysis of Category Ⅳ genes.**

Network visualization of enriched GO terms. Nodes represent individual GO terms, with sizes proportional to the number of enriched genes (Count). Edges between nodes denote functional similarity, while the color gradient reflects the statistical significance, represented by -log10 (Adjusted P-value).

**Supplymentary Figure 7 Expression patterns and allele frequency distribution analysis of candidate genes.**

(A) Relative expression levels (TPM) of candidate genes under Control and NaCl treatment conditions. Brown circles denote the Salt-Tolerant Population (STP), and green squares denote the Salt-Sensitive Population (SSP). (B) Allele frequency distribution analysis of the candidate genes between STP and SSP. The table lists the Gene ID, Analysis Window, total SNP Count, Significant SNP Count, Top SNP position and variant, Allele Frequencies in STP and SSP, and the corresponding *P* Values.
